# The dark side of centromeres: types, causes and consequences of structural abnormalities implicating centromeric DNA

**DOI:** 10.1038/s41467-018-06545-y

**Published:** 2018-10-18

**Authors:** V. Barra, D. Fachinetti

**Affiliations:** Institut Curie, PSL Research University, CNRS, UMR 144, 26 rue d’Ulm, F-75005 Paris, France

## Abstract

Centromeres are the chromosomal domains required to ensure faithful transmission of the genome during cell division. They have a central role in preventing aneuploidy, by orchestrating the assembly of several components required for chromosome separation. However, centromeres also adopt a complex structure that makes them susceptible to being sites of chromosome rearrangements. Therefore, preservation of centromere integrity is a difficult, but important task for the cell. In this review, we discuss how centromeres could potentially be a source of genome instability and how centromere aberrations and rearrangements are linked with human diseases such as cancer.

## Abstract (web summary)

Centromeres are the chromosomal domains that regulate assembly of the components required for chromosome separation. Here the authors review how centromeres are a potential source of genome instability and link centromere aberrations and rearrangements to human diseases such as cancer.

## Introduction

During each cell cycle, cells duplicate their genetic material before dividing it equally among daughter cells, so that the genetic information is faithfully distributed. To achieve this goal all eukaryotes have developed a common mechanism by which chromosomes are attached by microtubule fibers organized in a spindle that physically pull the two chromatids of the same chromosome towards opposite poles. Centromeres are key elements in this process. They are the site for the assembly of the kinetochore, the complex protein structure required for the interaction between spindle fibers and chromosomes, and for recruiting signaling components that ensure proper kinetochore-microtubule attachments (reviewed in ref. ^1^). They are also essential to maintain sister chromatids together by mediating assembly of the cohesin complex until proper chromosome separation occurs (reviewed in ref. ^2^).

With few exceptions, such as the point centromeres found in budding yeast, monocentric centromeres from plant to animal species are normally established on highly repetitive DNA arrays such as satellite DNAs and transposable elements, although the sequence itself is not conserved^3–5^. Human centromeres are built on a series of head-to-tail tandem repeats of 171 basepair (bp) AT-rich DNA^6,7^ named alpha satellites, that extend for several megabases (Mb) and make up ~3% of the genome (reviewed in ref. ^8^). Adjacent monomers can form higher-order repeat (HOR) units in which blocks of multiple repeats form a larger domain that can in turn be repeated thousands of times, giving rise to the Mb-sized human centromeres. Individual monomers show 50–70% sequence identity while HORs show up to 95% identity (reviewed in ref. ^8^). Pericentromeric DNA is also organized in tandemly and short repeated sequences, mainly containing Satellite I (chromosomes 3, 4 and the acrocentrics 13–15, 21, and 22), Satellite II (chromosomes 1, 2, 7, 10, 16, and 22) and Satellite III (chromosomes 1, 9, 10, Y and the acrocentrics 13–15, 21, and 22) (reviewed in ref. ^9^).

A fraction of centromere repeats contains a 17 bp motif named the CENP-B box. CENP-B boxes are the binding site for CENP-B (Centromere Protein B), the only sequence-specific centromeric DNA-binding protein identified so far^10^. The importance of CENP-B in centromere biology is currently a paradox. Its binding to DNA has been shown to play an important role in centromere function and chromosome segregation at least in mouse and humans^11,12^. Nevertheless, CENP-B and CENP-B boxes have not been identified outside vertebrates, with some rare exceptions in yeasts and insects. Furthermore, they are absent from neocentromeres and the male Y chromosome and CENP-B is not essential in mice (reviewed in ref. ^13^).

In addition, centromeric DNA sequence is not sufficient to maintain centromere position, but rather centromeres are epigenetically identified. From fission yeast to humans the histone H3 variant CENP-A was demonstrated to be the epigenetic mark for centromere identity and function (reviewed in ref. ^14^) by forming an unique centromeric chromatin in complex with the other canonical histones (H2A, H2B, and H4). CENP-A only marks active centromeres independently from DNA sequence^15^ and mediates centromere assembly through tightly regulated complex processes (reviewed in ref. ^14^).

Defects in any of the pathways that regulate centromere assembly and function can lead to chromosome mis-segregation and aneuploidy, common features of cancer cells (reviewed in ref. ^14^). However, in addition to inducing numerical chromosome alterations, centromere dysfunctions could also destabilize chromosome integrity, leading to structural alterations. In many cases, the centromere region itself is the site of chromosome breakage. This intrinsic fragility is probably due to the high density of repetitive sequences that makes the centromere more vulnerable and prone to rearrangements. In this review, we summarize the current knowledge on the types, causes, and consequences of alterations that lead centromeres to become potential sites of chromosome fragility and how these are linked with human diseases.

## Centromere breaks and disease

As mentioned above, failure in chromosome segregation leads to numerical and structural alterations, both common features of tumor cells^1^. Despite the fact that these alterations are known to participate in early steps of tumorigenesis and in cancer heterogeneity, the mechanisms underlying mitotic errors that lead to structural chromosome alterations still remain elusive. It is interesting to note that chromosome rearrangements and breaks often involve (peri)centromeric regions, with a frequency of up to 40–60% in certain cancer cell lines such as those deriving from colorectal carcinomas and adenocarcinomas (based on the analysis of 345 human carcinoma lines from the NCBI SKY/CGH database^16,17^). Different methods have allowed the identification of several cases of translocation involving centromeres in various tumors (Table 1)^18–33^ although the distinction between pericentromeric and centromeric regions is often hindered by the use of low-resolution technology and difficulties in aligning sequencing reads of repetitive DNA. For this reason, when related to breaks, the term “centromere” used in this review includes also its flanking heterochromatin.

**Table 1:** List of human tumors with the reported chromosome breaks around the centromere region

Chromosome involved	Type of tumor	Technique(s) used	Reference
18, 8, 7	Breast cancer	FISH, aCGH	Alsop et al.^18^, Cooke et al.^19^, Bièche et al.^26^
1, 18	Melanoma	FISH	Alsop et al.^18^, Smedley et al.^27^
18	Colorectal	FISH	Alsop et al.^18^
18, 8	Pancreatic	FISH, aCGH	Alsop et al.^18^, Cooke et al.^19^
Several	Squamous cell carcinoma	FISH, SKY, aCGH	Hermsen et al.^28^
Several	Adenocarcinoma	FISH, SKY, aCGH	Hermsen et al.^28^
Several	Oral squamous cell carcinoma	FISH	Hermsen et al.^29^
Mostly 3, 5	Head and neck squamous cell carcinoma	FISH, fiber-FISH, aCGH, MLPA	Martinez et al.^30^, Martins et al. ^31^
1	Non-Hodgkin’s lymphoma	FISH	Baccon et al.^32^
1	Multiple myeloma	FISH	Baccon et al.^32^
1	Ovarian cancer	FISH	Thompson et al.^33^
10, 14, 7, 21	Cervical squamous cell carcinoma	FISH	Backsch et al.^20^
Mostly 17	Hematologic malignancies	FISH	Adeyinka et al.^21^
Several	Prostate cancer	G-banding, SKY	Balachandar et al.^22^
17	Male breast cancer	MLPA, FISH	Lacle et al.^23^
1, 8	Hepatocellular carcinoma	Whole genome sequencing, aCGH	Fernandez-Banet et al.^24^
1, 7	Acute myeloid leukemia	FISH	Ganly et al.^25^

*FISH* fluorescence in situ hybridization, *f-FISH* fiber FISH, *aCGH* array-based comparative genomic hybridization, *SKY* spectral karyotyping, *MLPA* multiplex ligation-dependent probe amplification, *G-banding* Giemsa banding, *WGS* whole-genome sequencing

A functional genomic analysis on 8000 human cancer genomes from high-resolution array-CGH platforms present in the GEO database revealed that whole-chromosome arm gain, loss, or translocation are in fact common alterations in tumors^34^. Altogether, this suggests that centromeres are indeed fragile and prone to rearrangements during tumorigenesis.

### Structural consequences of centromere breakage

**Fig. 1:**
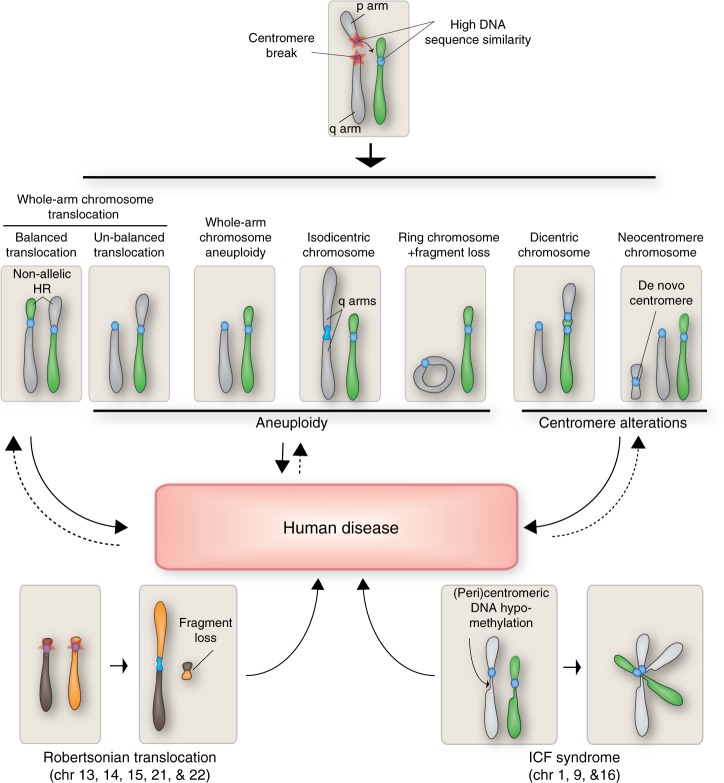
Centromere breakage and human diseases. Representation of the genomic alterations deriving from centromere breaks such as aneuploidy, dicentric, and neocentromere chromosomes, Robertsonian translocations together with loss of methylation at (peri)centromere (typical of ICF syndrome), all known to contribute to human diseases

Centromere breaks can generate whole-arm chromosome translocations in which the whole-arm of a chromosome fuses to another chromosome with either no loss of genetic information (balanced translocation) or with loss of genetic material and generation of a derivative chromosome (unbalanced translocation) (Fig. 1). One potential mechanism for the formation of whole-arm chromosome translocations are non-allelic exchanges in non-homologous chromosomes (Non Allelic Homologous Recombination, NAHR) at centromeric repeats during DNA repair of the break. This recombination event between homologous regions located at different loci on different chromosomes might be driven by the high DNA sequence similarity between centromere repeats that favors the occurrence of NAHR. This type of recombination event has been frequently observed in samples from couples with balanced reciprocal translocations during a study for preimplantation genetic diagnosis. Here, it was observed that the centromere itself was the actual breakpoint of the translocation in the majority of analyzed individuals^35^, suggesting that damage at this site is not so rare. The result of these translocations was the appearance of derivative chromosomes with two centromeres very close to one other often with mixed and different lengths of alpha-satellite repeats. Occasionally, more than one chromosome was involved in this breakage at the same time and, in some cases, the derivative chromosomes were conserved in the family, indicating that they can be meiotically and mitotically stable.

Centromere breaks and translocations can also induce the formation of isochromosomes, chromosomes that carry identical copies of the same arm joined through a single centromere, therefore generating partial trisomy (Fig. 1). It was observed that the smaller the arm, the more likely the survival of the embryo, probably due to the reduced amount of duplicated genetic material. Two main molecular mechanisms have been suggested to generate isochromosomes. The first one described is a phenomenon called “centromere mis-division”: instead of dividing longitudinally, the chromosome would undergo a transverse splitting by separating p- and q- arms^36^. However, most isochromosomes are rarely monocentric, but isodicentric with two centromeres very close to one another (see “Causes and consequences of dicentric chromosomes” paragraph). The second mechanism known as U-Type exchange, an event that takes place when two sister chromatids undergo a double-strand break and the two broken ends fuse creating a dicentric chromosome^37,38^. Wolff et al. demonstrated that the majority of breakpoints resulting in isochromosome X formation—the most common isochromosome, responsible for some cases of Turner syndrome—involved the pericentromere and not the centromere itself^39^. They also observed that it is the proximal short arm that is prone to break and re-join the sister chromatids or the homologous X chromosomes via U-Type exchange. However, more recent characterization of the pericentromeric region of chromosomes highlighted the presence of inverted repeats that could be subject to NAHR giving rise to isochromosomes^40–42^. Other types of replication-dependent recombination mechanisms such as FoSTeS (Fork Stalling and Template Switching) and MMBIR (microhomology-mediated break-induced replication) could also promote isochromosome formation^41^.

### Centromere breaks in disease

Several tumor types that exhibit chromosomal breakpoints occurring at centromeric regions with high frequency are also associated with high degree of chromosomal instability (CIN) (Table 1). For example, the cytogenetic feature of oral squamous cell carcinoma is the high presence of isochromosomes and unbalanced whole-arm translocations that originate from chromosomal breakpoints. These chromosomal alterations with consequent loss of genetic material mainly occur at centromeric regions (134 out of 218 chromosomal breaks, >60%) and involve several chromosomes including acrocentrics^29^. Similarly, head and neck squamous cell carcinoma is characterized by the presence of whole-arm translocations which exhibit breakpoints close to the centromere (84%)^30^. This high percentage suggests that centromeric breaks are directly involved in the origin and evolution of this tumor type although, even in this case, we cannot discriminate whether centromere breakage is a direct cause or simply a consequence of high levels of rearrangements. Indeed, data collected from snapshots of late stage cancer tissues cannot really tell us when such abnormalities occurred during tumorigenesis. Some specific alterations characterize other cancer types such as gain or loss of 1q, 2p, 6p, or 16q arm in retinoblastoma^43^ and correlate with the degree of malignancy and prognosis^21^. Hematologic malignancies also show a high frequency of the isochromosome i(17)q, whose breakpoint maps in a complex pericentromeric region characterized by palindromic LCRs separated by a spacer, which would support the hypothesis that the isochromosomes are originated by NAHR^40^.

Centromere alterations and breaks have also been found in human genetic diseases like the Immunodeficiency, Centromeric region instability, Facial anomalies syndrome (ICF), where mutations of DNA Methyl-Transferase 3 Beta (DNMT3B) (55% of reported cases), ZBTB24, CDCA7, or HELLS^44,45^ correlate with loss of DNA methylation, pericentromeric breaks, and rearrangements near the centromere with consequent whole-arm deletion (Fig. 1). The exact molecular mechanisms that lead to these defects are still unknown, but previous reports suggest a link between defects in DNA replication and/or chromosome segregation and DNA hypomethylation. Indeed, reduced DNA methylation at centromere/pericentromere has been correlated with aneuploidy^46^ and with accumulation of micronuclei that contain centromere-positive fragments in human lymphocytes with global hypomethylation^47^.

Altogether, these evidences suggest that a significant fraction of chromosome instability typically observed in cancer potentially arises from centromeric breakage. Martinez and van Wely described how most of the copy number variations (genetic dosage imbalance) observed in cancer correspond to entire chromosomes or chromosome arms, suggesting that the (peri-)centromere is the preferential break site^48^. Thus, an initial centromere break could make chromosome segments more prone to several rounds of breakage, therefore directly contributing to cell heterogeneity during cancer transformation. This “centromeric fission model” would explain the high degree of chromosome alterations typically observed in tumors. Given that tumors exhibit both centromere breaks and aneuploidy, it would not be too rash to think that they arise from a common mechanism and that centromere breaks drive a fraction of the CIN observed in cancer.

## Causes of centromere breakage

The mechanisms that lead to centromere breaks are still not understood. Below we summarize the current state of knowledge on the possible sources of centromeric ruptures.

### Chromosome mis-segregation

**Fig. 2:**
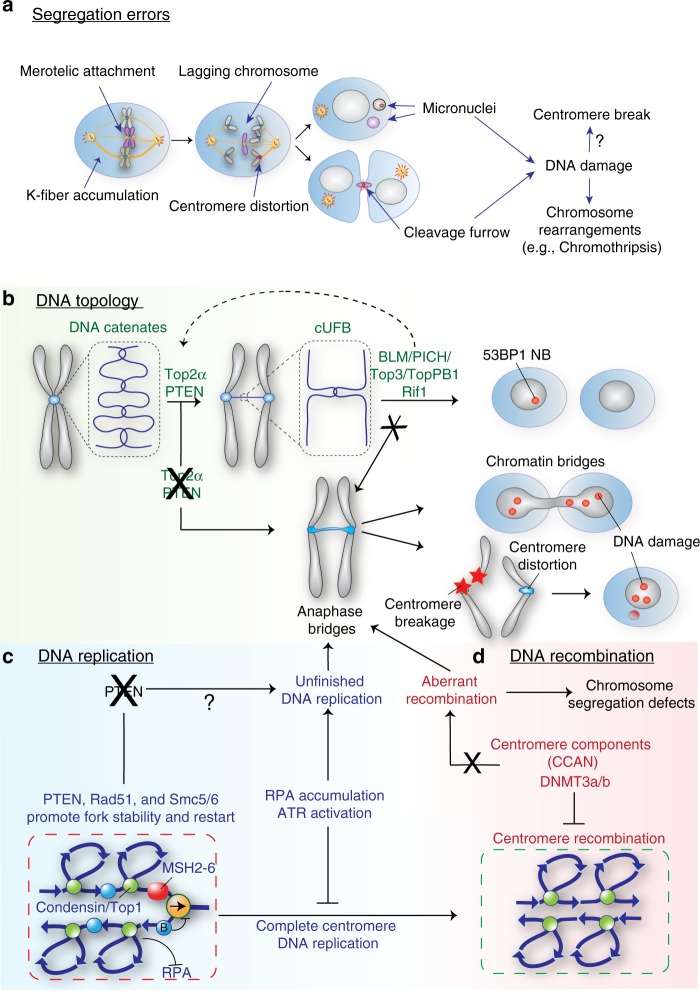
Origins of centromeric breaks. Schematic of the possible causes that lead to chromosome breaks at centromere. **a** Segregation errors: merotelic attachment and k-fiber accumulation are the sources of lagging chromosome formation and centromere distortion in mitosis leading to micronuclei and centromere breaks. **b** DNA topology: the complex DNA topology enriched in catenanes is normally resolved by topoisomerases prior to completion of sister chromatid separation. Resolution of UFBs is also crucial for maintenance of genome integrity in which a fraction of those results in 53BP1 nuclear bodies (NB) that are repaired in the next cell cycle. Alterations in the centromere decatenation pathway can lead to anaphase bridges and DNA breaks. **c**, **d** DNA replication and recombination: due to its highly repetitive sequences a non-canonical pathway of DNA damage and homologous recombination proteins is implicated in the correct completion of centromere replication in order to avoid formation of anaphase bridges, unscheduled recombination and, consequently, segregation errors and breaks

Chromosome segregation errors themselves are one of the proposed potential sources of structural chromosomal alterations and centromere breaks. For example, increased incidence of lagging chromosome formation was linked to the accumulation of DNA damage markers at the cleavage furrow, activation of the ATM/Chk2 response and to an increasing fraction of structural chromosome alterations^49^ (Fig. 2a). Lagging chromosomes are generally caused by merotelic attachments in which a single kinetochore is attached to microtubules arising from both spindle poles, forcing a single chromatid into a tug-of-war between both poles^50^. Intriguingly, a more recent study revealed a mitotic role for ATR in preventing lagging chromosomes^51^. ATR is normally activated by persistent single-strand DNA originated by stalled replication forks or DNA damage. In mitosis ATR localizes at centromeres through Aurora A and CENP-F where it binds RPA-positive-ssDNA generated by R-loops (DNA-RNA hybrids) at centromeres. Here it phosphorylates and activates Chk1 which in turn activates Aurora B, necessary to correct errors in the microtubules–kinetochore attachment^1^. What generates RNA loops at centromeres and if they contribute to the overall centromere stability together with ATR is unknown, but the presence of ATR and RNA loops reinforces the concept that centromeres are potential fragile regions that need a dedicated surveillance mechanism.

Mis-segregated chromosomes can also be encapsulated in micronuclei, which are a source of genome instability. Indeed lagging or mis-aligned chromosomes generated by microtubule depolymerizing drugs or selective centromere inactivation, respectively, are encapsulated in micronuclei, where they undergo chromosome shattering and re-shuffling, a phenomenon known as chromothripsis^52^. However, the fate of centromeric regions during the process of chromothripsis was not determined, likely due to the current difficulty of mapping these repetitive regions by conventional DNA sequencing. A direct correlation between centromere breaks and chromosome mis-segregation was proposed to exist upon alteration of the mitotic spindle^53^. Mouse embryonic fibroblasts (MEFs) depleted of Dido3—structural component of the spindle pole—or human cells carrying a mutation in the tumor suppressor APC that regulates microtubules and kinetochore attachment, accumulate lagging chromosomes, distorted centromeres/kinetochores and eventually micronuclei with γH2AX foci (an early DNA damage marker) in proximity to or at centromeric regions (up to 75%). The authors of these studies propose that the accumulation of kinetochore fibers (K-fibers) and loss of spindle control are the sources of these breaks (Fig. 2a). However, it is still under debate if microtubules can generate enough force to break the double helix.

Further studies are needed to demonstrate a direct causal effect between chromosome mis-segregation and the formation of centromere breaks. Without doubt the centromeres are subjected to high level of mechanical stress during chromosome segregation due to microtubules pulling on the centromere/kinetochore platform. It is tempting to speculate that, to support this stress the centromeres have evolved to accumulate long stretches of repetitive sequences that could form a unique DNA architecture.

### Centromere topology

Due to the repetitive nature of their DNA sequences, it is likely that repetitive centromeres have a highly complex DNA topology, which may lead to accumulation of DNA catenanes and formation of DNA loops between their DNA repetitive arrays. Both DNA catenanes and loops represent perfect substrates for the DNA recombination machinery and DNA topoisomerases thus making centromeres potential “fragile” regions in the genome. Pioneering studies in flies^54^ or using human BACs inserted in *Xenopus*^55^ first described the architecture of centromeres. In *Drosophila* *melanogaster*, in vitro studies using nuclear magnetic resonance, circular dichroism, and mass spectrometry show complex and stable structures at centromeres called i-motifs^54^. These are four-stranded structures formed by the association of two duplex DNA strands that are organized in an antiparallel way, reinforced by the occurrence of hemi-protonated C:C^+^ base pairs. Despite the fact that the specific region analyzed in this study (the G-rich dodeca satellite) was recently found not to be enriched with CID^56^ (CENP-A homolog in flies), this data suggests that at least regions surrounding functional centromeres (generally marked by CID) contain specific secondary structures. Likewise, using electron microscopy on a BAC containing mainly centromeric DNA from chromosome 17, Aze et al. observed the presence of positive supercoiled DNA loops following incubation with *Xenopus* egg extract^55^. These stable structures were proposed to be replication- and condensation-dependent, since full proteinase K treatment led to the disappearance of DNA loops. Indeed, mass spectrometry analysis on reconstituted centromeric BAC revealed the presence of subunits of the condensin complex and topoisomerase II (Top2). The enrichment of Top2 supports previous in vitro data that showed a Top2-mediated DNA cleavage activity on oligonucleotide-forming hairpins of alpha-satellite DNA^57^.

During mitosis centromeric DNA strands intertwine as a natural consequence of DNA replication, causing an accumulation of catenanes at centromeric regions. At early stages of mitosis, these structures play a role in preventing premature sister chromatid disjunction^58^, but during anaphase they need to be resolved to preserve centromere stability (Fig. 2b). This catenated DNA also leads to the formation of a particular class of nucleosome-free DNA bridges defined centromeric ultra fine bridges (cUFBs), not detectable with DAPI or other usual DNA staining^59^. The origins and the role the of this type of centromeric UFBs are still unclear, but it was suggested that they are physiological structures that arise from unresolved topological domains and/or recombination intermediates issuing from potential DNA loops at centromeric regions^60^.

DNA decatenation is mainly performed by Top2a and its inhibition leads to an increased number of UFBs and chromosomal segregation defects^59^ (Fig. 2b). In support of its role in DNA decatenation at (but not only) centromeric regions after DNA replication, Top2a accumulates at centromeric regions in budding yeast^61^ and was shown to be required to resolve topological domains at converging forks also at centromeric regions^62^. Top2a function at UFBs is supported by two helicases: Bloom’s syndrome protein (BLM) and Plk1-interacting checkpoint helicase (PICH), which both decorate UFBs^59,63^ (Fig. 2b). PICH is recruited at cUFBs in prometaphase and is necessary for BLM localization^64^. In addition, PICH has been shown to have a nucleosome remodeling activity required to remove histones from UFBs. This function was proposed to give a sufficient temporal window for Top2a to easily access DNA catenates, and, in turn, to decatenate sister chromatids DNA^64^.

BLM and PICH are essential for correct chromosome segregation. Indeed, their depletion is associated with changes in centromeric structure, highlighted by a reduced signal of centromeric probes by FISH and by an accumulation of denser centromeric chromatin observed by electron microscopy^65^. PICH and BLM loss also correlates with non-disjunction of sister chromatids, formation of micronuclei, and lack of recruitment of Top2a^65^. Finally, BLM is also required to recruit other components to UFBs critical for their resolution such as Topo 3α, that in turn stimulates BLM activity^66^. Interestingly, Top3 together with Rqh1 (a RecQ helicase) was demonstrated to regulate CENP-A^Cnp−1^ binding at centromere in fission yeast possibly regulating the interplay between topology, recombination, and transcription^67^. Other factors implicated in the resolution of c-UFBs are TopBP1^68^ and Rif1^69^ that either control Top2 activity, or directly promote c-UFB resolution, respectively.

In summary, a correct regulation of the “centromere decatenation pathway” is critical for the maintenance of genome stability, since any defects in this process lead to segregation errors, chromosome breaks and rearrangements (including at centromeric regions) and hence, potentially, to diseases. One example of disease is Bloom’s syndrome, an inherited disorder characterized by short stature, sun sensitivity and a strong predisposition to any kind of cancer. This syndrome is caused by mutations of BLM that leads to sister chromatids exchanges (SCE), a marker for increased homologous recombination (reviewed in ref. ^70^).

### DNA replication

Unfinished or incorrect DNA replication is another possible source that contributes to centromere breaks. Correct replication of the genome is a prerequisite for the maintenance of genome stability. The licensing of DNA replication origins is restricted to one round per cell cycle by cyclin-dependent kinases, so that the replicative apparatus cannot reinitiate a new synthesis session in the same cell cycle. Along the same line, the presence of only one centromere per sister chromatid is guaranteed to ensure the faithful segregation of chromosomes (see “Causes and consequences of dicentric chromosomes” paragraph). Indeed, induction of centromere re-replication in budding yeast was shown to alter chromosome segregation and induce aneuploidy by destroying the symmetry of normal sister chromatids leading to bipolar attachments of the same chromatid^71^.

Because of the centromeres’ repetitive nature, their replication might represent a demanding job that generally makes this region unstable, prone to replication errors and to recombination due to the formation of secondary structures (reviewed in ref. ^72^). How higher Eukaryotes deal with replication problems at centromeres is still unknown. Recent studies on reconstituted chromatin of BACs containing human centromeric DNA in *Xenopus laevis* showed that centromeric regions display similar replication patterns to other repetitive-free DNA regions. However, centromere DNA was enriched in DNA repair factors such as MRE11/RAD50, Ku80, PARP1, and the MSH2-6 complex (involved in mis-match repair) suggesting that these factors are necessary for correct centromere replication^55^. Interestingly, following replication stress by aphidicolin treatment, centromeric regions were shown to be depleted of the ssDNA-binding protein RPA-1 (normally enriched under this circumstance) and, consequently, the ATR pathway was not activated. This checkpoint silencing, likely mediated by the positive supercoiling of centromeric DNA structures, together with an increased activity of the MSH2-6 complex, was proposed to be required for successful finalization of DNA replication at centromeric regions^55^ (Fig. 2c).

Curiously, a direct link between the centromeric component CENP-B and DNA replication has been shown. CENP-B has been shown to take part in centromere replication preserving its integrity during replicative fork stall in both yeast and humans. In fission yeast CENP-B homologs (autonomously replicating sequence-binding protein 1, Abp1, and CENP-B homologs 1 and 2, cbh1/2) were shown to induce the silencing of long terminal repeat (LTR) retrotransposons, recruitment site of Sap1, in order to prevent replication stress by making the forks progress^73^. In humans, alpha-satellite sequences seem to behave like origins of replication^74^ and CENP-B, which binds these sequences, is required to regulate the assembly of the replication apparatus on these origins. Consequently, CENP-B depletion results in a change of alpha-satellite chromatin that leads to an increased number of replication origins at centromere^74^. These evidences would suggest that beside its role in centromere function^12,75^, CENP-B binds the CENP-B boxes in the alpha-satellite repeats in order to preserve centromere integrity by regulating DNA synthesis and replication fork stall.

The presence of repetitive sequences per se might not be the only problem that replication forks have to deal with at centromeres. For example, during DNA synthesis of the non-repetitive centromere of budding yeast that contains a defined sequence of 125 bp enriched with AT, replication forks pause while approaching centromeric DNA, due to the presence of the centromeric protein complex^76^. Additionally, at least half of centromeric regions in budding yeast were identified as sites of converging forks, therefore generating even more steric impediment for fork progression^62^. If not solved correctly, the ensuing replication termination sites can be the source of DNA breaks as observed in the topoisomerase top2 mutant, further demonstrating how centromeres can potentially become fragile sites.

DNA replication stress seems to be one of the main causes of chromosomal instability in some colorectal cancers. Silencing of the so called “CIN suppressor genes” (such as PIGN, MEX3C, and ZNF516) in this type of tumors drives to a variety of chromosomal instability and segregation defects (e.g., lagging chromosomes, acentric fragments, anaphase bridges, chromosome fusion leading to dicentric chromosomes)^77^. These abnormalities that lead to DNA damage can be rescued by the addition of nucleosides, strongly suggesting the involvement of replication stress in the generation of this chromosomal instability. Replication stress induced by aphidicolin was also shown to lead to enrichment of double-strand breaks (DSBs) at centromeric regions in several cancer cell lines^78^. This intrinsic fragility might also be enhanced by the fact that, at least in humans, centromeres are late replicated regions^79^ and therefore, under certain conditions, they may be more prone to stall or incomplete DNA replication, with consequent emergence of UFBs and/or genome instability.

A wide range of other solid tumors which exhibit CIN also have mutations in PTEN, a tumor suppressor gene involved in the repression of phospoinositide 3-kinase (PI3-K) signaling cascade. Interestingly, PTEN was shown to bind centromeres by interacting with CENP-C through its C-terminus^80^. PTEN centromeric localization is important to prevent centromere breakage and to maintain chromosome stability^80^ possibly by regulating homologous recombination (HR) during the recovery of replication fork stall, via Rad51 recruitment^81^. Additionally, PTEN has also been correlated with sister chromatids decatenation, a process occurring after DNA replication and essential for proper chromosome segregation (see section: “Centromere topology”). Indeed, PTEN C-terminus physically binds and regulates DNA topoisomerase IIα (TOP2A) expression in a p53-independent manner by stabilizing TOP2A via its deubiquitination through OTUD3^82^. In summary, by associating with centromeric regions via CENP-C, PTEN is involved in the resolution of replication fork pausing by promoting HR and in regulating DNA decatenation via TOP2A (Fig. 2b, c). This would explain why tumors that suppress PTEN function exhibit chromosome instability.

In conclusion, replicating centromeric DNA is a difficult task for the cells due to the accumulation of large regions of repetitive DNA. Repetitive sequences are generally unstable and might form secondary structures that could induce replication fork stalling and high levels of recombination. This, in turn, could lead to DNA breakage in certain genetic backgrounds. In this view, it is not surprising that centromeres evolved to have a unique replication apparatus rich in DNA repair factors normally required for DSB processing and repair.

### Heterochromatin and recombination

In vertebrates, heterochromatic regions surround centromeres in which the chromatin assembly factor CAF-1 deposits H3 and promotes H3K9 mono-methylation. This is in turn recognized by the histone-lysine N-methyl-transferase Suv39h1 which tri-methylates H3 on Lysine 9 (H3K9m3). H3K9m3 creates the docking sites for the heterochromatin protein 1 (HP1) complex, whose recruitment is essential for heterochromatin formation at those peri-centromeric regions (reviewed in ref. ^83^). Interestingly, in fission yeast pericentromeric heterochromatin is characterized by the constitutive presence of low levels of γH2AX^84^, typically associated with DNA damage. This intrinsic stress might be caused by continuous stalling of replication forks. In support of this idea, both the SMC5/6 and Brc1, that binds γH2AX, are enriched at the centromere to ensure replication fork restart^85,86^.

It has been established that stalled replication forks are mainly resolved through homologous recombination. Unresolved HR intermediates during replication fork re-start lead to replication errors, which in turn lead to deleterious consequences during chromosome segregation such as anaphase bridges and centromere breaks. Centromere repeats provide a substrate for HR by promoting break-induced replication (BIR) even in the absence of stress conditions^87,88^. In *C. albicans* it was proposed that the main HR regulators Rad51 and Rad52 control centromere fork stall and restart by directly controlling CENP-A deposition since it acts as a physical barrier for fork progression^89^. It has to be tested if a similar mechanism exists in higher Eukaryotes.

This suggests that recombination is an essential event in centromere maintenance, although it might expose centromeres to potential chromosome rearrangements. The heterochromatic state of the pericentromere region itself is necessary to maintain centromere integrity by protecting it from high levels of recombination. Indeed, defects in heterochromatin formation correlate with gross chromosomal rearrangements and segregation defects as observed in fission yeast^90^. In mouse it has been shown that an increase in recombination at centromere leads to changes in the length of the repeats, that in turn affects centromere integrity^91^. Moreover, loss of DNA methylation at mouse minor satellite repeats due to the absence of DNA methyl-transferases 3A/3B, leads to a deregulation of recombination at centromere and shortening of centromere repeats^91^ (Fig. 2d). Mechanistically, how DNA methylation is involved in centromere organization, function and stability has not been fully explored and remains an open question. We can speculate that either the modified epigenetic status of chromatin at minor satellites following DNA methylation loss or the shortening of centromeric repeats, or both events, may affect the binding of centromeric proteins, thus resulting in genomic instability. Finally, it has been recently shown that gradual removal of CENP-A and some of its associated components such as CENP-C/T/W from human centromeres correlates with a drastic increase in centromere aberrations observed by cen-CO-FISH (centromere chromosome orientation fluorescent in situ hybridization), where more than 90% of centromeres per metaphase displayed abnormal patterns of the centromeric probes^92^ (Fig. 2d). In addition, CENP-A loss led to excision of centromeric repeats, assessed by the presence of centromere probes outside the centromeres^92^.

To which extent recombination at centromere/pericentromere is beneficial for centromere is unclear. Centromeres are highly dynamic regions whose size and diversity acquired during evolution could be explained by recombination. However, it is well-known that recombination at centromere is normally repressed during meiosis as demonstrated by the fact that the crossing over between homologous chromosomes is suppressed (so-called “recombination cold spots”) (reviewed in ref. ^93^). This suppression mechanism is necessary since recombination would affect chromosome cohesion and/or correct kinetochore attachment to fibers radiating from spindle poles, and in turn could generate aneuploid nonviable gametes (reviewed in ref. ^94^). Nevertheless, it has also been observed that the occurrence of homologous recombination between centromere repetitive sequences leads to covalently closed loops and that these structures can have an important role in the establishment of a functional centromere^60^. In addition, non-crossover gene conversion has been shown to occur at centromere probably promoting sequence exchanges^95^. The importance of recombination within centromeric DNA repeats needs to be further demonstrated, in particular in light of the existence of the stably inherited neocentromere that can form at non-repetitive regions^96^.

## Neocentromere implication in genome instability and disease

The neocentromere is a rare and novel class of centromere, described in humans and other species, that normally lacks repetitive DNA sequences and their binding partner CENP-B (reviewed in ref. ^97^). Due to the absence of alpha satellites, neocentromere formation was among the strongest evidence in support of the centromere being epigenetically defined. Neocentromeres appear in chromosomal locations different from the original centromere and usually form in euchromatic and non-repetitive regions, with some exceptions located in heterochromatic regions such as the ones in the Y chromosome (reviewed by ref. ^98^) and the repetitive L1 long interspersed nuclear elements (LINEs)^99^. Interestingly, evolutionary studies suggest the existence of recurrent hotspots for neocentromere seeding, suggesting that centromeres are often re-used after they have been inactivated in ancestral species (reviewed in ref. ^100^) and might acquire repetitive sequences over time. Curiously, the majority of neocentromeres artificially formed by centromere depletion in chicken cells and *C. albicans* arise close to the original centromeres^101,102^ regardless of their chromatin state^102^. This suggests that CENP-A accumulates also at pericentromeric regions forming so-called “CENP-A clouds” around the centromeres^103^. In few other cases, neocentromeres formed at other repetitive sequences such as telomeric regions similar to that described in fission yeast^104^.

### Neocentromere and disease

**Fig. 3:**
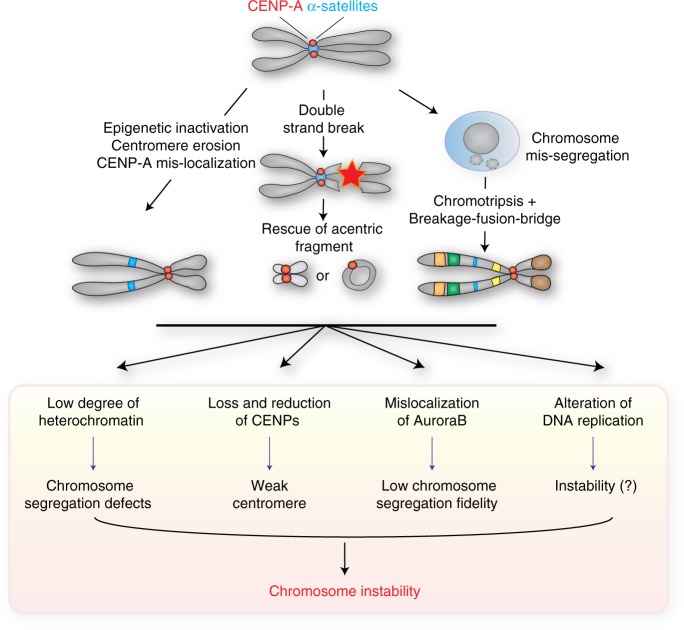
Neocentromeres and chromosome instability. A neocentromere forms at chromosomal locations different from the centromere’s original position. The possible causes of neocentromere formation, depicted here, span from epigenetic inactivation, centromere erosion, CENP-A mislocalization to rescue of acentric DNA fragments following chromosome rearrangements. Neocentromeres display, however, a low degree of heterochromatin, loss and reduction of centromeric proteins (CENPs), mislocalization of Aurora B and alteration of DNA replication that cause a decrease in chromosome segregation fidelity

It is still unclear if there is a selective advantage in forming a neocentromere. Neocentromere formation could play a role in maintaining the correct chromosome content in the case of centromere inactivation or loss. Despite this positive role, neocentromere formation is generally associated with cancer or genetic dysfunction (reviewed in ref. ^97^). Indeed, since their first observation in 1993 during the routine karyotyping of a boy with learning difficulties^96^, several different constitutional human neocentromeres have been described in individuals with congenital abnormalities, development delay and intellectual disability^97^. Up to now little is known about the molecular mechanisms that lead to neocentromere formation (Fig. 3). Certain cancers bear neocentromeres, suggesting that chromosome rearrangements may be one of the driver events for de novo centromere formation. Indeed, neocentromere formation was hypothesized to be a mechanism that cancer cells use to rescue the acentric chromosomal fragments arising from their gross chromosomal rearrangements to allow cell proliferation (reviewed in ref. ^105^). This hypothesis might also be sustained by the fact that cancer cells continuously undergo genomic rearrangements that could also potentially affect the function of the native centromere. However, the correlation between neocentromeres and cancer may be severely underestimated since cancer cells’ karyotype is rarely analyzed with respect to centromere regions using alpha-satellite FISH.

For example, neocentromere formation was correlated with the generation of small supernumerary marker chromosomes (sSMCs; the presence of a structurally abnormal 47th chromosome in the genotypic makeup) in 3% of published sSMC cases (reviewed in ref. ^97^). How and when a neocentromere is formed is still a matter of debate and much still needs to be characterized. The most common rearrangements involving centromeres are inverted duplications and interstitial deletions. Both originate from chromosomal breaks that in the first case generate a chromosome fragment without a centromere and with an inverted duplication. In interstitial deletions, chromosomal breaks lead to the formation of a ring chromosome and the presence of an additional acentric or centric fragment depending on the site of deletion (paracentric or pericentric respectively) (reviewed in ref. ^97^). In contrast to other circular chromosomal rearrangements such as double minutes (chromosome fragments carrying gene amplification), these ring chromosomes resulting from interstitial deletions can acquire a neocentromere. For example, it was observed that a patient with intellectual disability, brachyphalangy and growth hormone deficiency carried a 20 Mb deleted region present, as a mosaicism, in a supernumerary ring chromosome stabilized by the neocentromere at 13q31-q32^106^. However, in vitro observations showed that ring chromosomes are prone to mis-segregation regardless the presence of the neocentromere^106^.

When neocentromeres form to rescue inverted duplicated fragments^107^, the resulting cell will be partially tetrasomic if the fragment segregates together with the intact chromatid; alternatively, if the fragment segregates together with the deleted chromatid, the cell will be partially trisomic. Indeed, neocentromere presence is associated with partial monosomy, trisomy, tetrasomy or ring chromosomes. These aberrations are in turn accompanied by developmental delays, short stature, digital malformations, facial dysmorphisms, renal defects and Turner-like syndrome^106,108–111^. In addition, some tumors such as liposarcoma, retinoblastoma, non-Hodgkin’s lymphoma, acute myeloid leukemia and lung cancer show complex rearranged chromosomes with the presence of neocentromeres^112–116^. For example, in some cases of liposarcoma neocentromeres are markers of tumor-like SMC rings and giant rods containing the amplification of the 12q1415 region^117^, or the alphoid-deprived inverted 10q duplication and the chromosome 8-derived ring chromosome in acute myeloid leukemia^118^.

Neocentromeres have also been associated with gigantic (up to 600 Mb) supernumerary accessory chromosomes (or neochromosomes) typically observed in well-differentiated/dedifferentiated liposarcoma (WD/DDLPS). Studies that followed the evolution of five of these neochromosomes show evidence of chromothripsis, after several cycles of breakage and re-ligation of chromosomes (breakage fusion bridge, BFB), centromeric corrosion followed by de novo centromere formation and telomere capture^119^. This process of centromere erosion with loss of up to 78% of α-satellites and surrounding pericentromeric DNA observed in these WD/DDLPS cells could be one of the first events that lead to neocentromere establishment. Indeed, this reduction of α-satellite DNA was also observed in other cases of less rearranged chromosomes containing a neocentromere^15,120^.

### Instability of chromosomes containing neocentromeres

This correlation between chromosome alterations and neocentromeres can be envisaged in either direction, with neocentromeres themselves potentially representing a source of genomic rearrangements (Fig. 3). Indeed, a neocentromere is characterized by a lower degree of heterochromatin around CENP-A binding sites with respect to a constitutive centromere^121^. This peculiarity seems to be correlated with the high frequency of cohesion defects at neocentromeres that, in turn, affect chromosome segregation. Importantly, the absence of CENP-B, another feature of neocentromeres, has been correlated with reduction of centromeric CENP-C and consequent increase of errors during chromosomal segregation^75^. Additionally, an example of neocentromere present on chromosome 4^15^ displays mislocalization of Aurora B, an essential protein for the error-correction of kinetochore-microtubule attachment^122^. A further characterization of neocentromere-containing chromosome is required to determine if other molecular mechanisms contribute to its instability.

Neocentromere formation is also believed to change the timing of DNA replication around its chromosomal area, although the function and consequences of this alteration are still not clear. Studies on the well-characterized neocentromere of chromosome 10-derived marker chromosome [mardel(10)] showed that neocentromere formation correlates with a delay in the replication timing of the area surrounding its location^123^. Consistently, artificially generated neocentromeres in chicken cells^101^ or in the yeast *C. albicans*^124^ lead to a change in replication timing initiation of the chromosomal domain that hosts the de novo centromeres (from early to late in chicken and from mid/late to early in yeast). These results suggest that the formation of a new centromere affects the initiation of DNA replication for reasons that still need to be investigated. It is possible that late replication timing of centromeres in vertebrates such as the one observed in humans^79^ is a requisite for centromere function and/or stability.

### Artificial neocentromere formation

Many methods have been devised to induce neocentromere formation using several model systems, in order to understand how they arise (for space constraints we will not discuss experiments in plants, but we recommend this review on the topic^125^). Using *Drosophila* *melanogaster*, Williams et al. discovered that acentric mini-chromosomes acquire neocentromere activity following chromosome breaks induced by γ-irradiation, although the presence of centromeric proteins was never tested^126^. In a follow-up study using a supernumerary minichromosome screening in flies after irradiation, Maggert and Karpen observed neocentromere formation only in fragments juxtaposed to an active centromere^127^. A further link between DNA damage and neocentromere formation came from experiments done in human cells in which transiently overexpressed CENP-A was shown to bind sites of DSBs^128^. This report, together with the identification of CENP-A chaperone HJURP at sites of DNA damage^129^, from that originates its name, Holliday Junction Recognition Protein—suggests that sites of DSBs bound by CENP-A might initiate neocentromere formation as a mechanism to prevent the loss of otherwise acentric chromosomes.

Neocentromeres could also be formed by transient overexpression of CENP-A in flies without evidence of DNA damage^130^ where telomeres and heterochromatin regions are shown to be hotspots for CENP-A islands and de novo centromere formation^131^. With the exception of budding yeast that harbors a point centromere, neocentromeres can also be induced in fungi by the deletion of the native centromere using a homologous recombination strategy in *C. albicans*^102,132^ or the Cre-Lox system in fission yeast^104^. In human cells overexpression of CENP-A does not lead to neocentromere formation, with the exception of overexpression of its variant constitutively ubiquitylated on K124R-Ub^133^, although the function of this CENP-A ubiquitination is controversial^134^.

In some rare cases, other neocentromeres have been reported in an apparently unarranged chromosome^15,120,135^. It has to be pointed out that cytogenetic analysis is rarely performed in a healthy population, therefore these “rare cases” of neocentromeres may be underestimated. During neocentromere formation in an apparently unarranged chromosome, the functional centromere has been shifted to a new position along the chromosome arm forming a neocentromere, while the original centromere becomes inactive. This configuration of a neocentromere formed on a chromosome that still harbors alphoid DNA is called a pseudodicentric chromosome. Centromere inactivation is required to prevent the formation of a chromosome with two functional centromeres named dicentric chromosome. The mechanisms via which one of the centromeres is inactivated are mainly uncharacterized.

## Causes and consequences of dicentric chromosomes

**Fig. 4:**
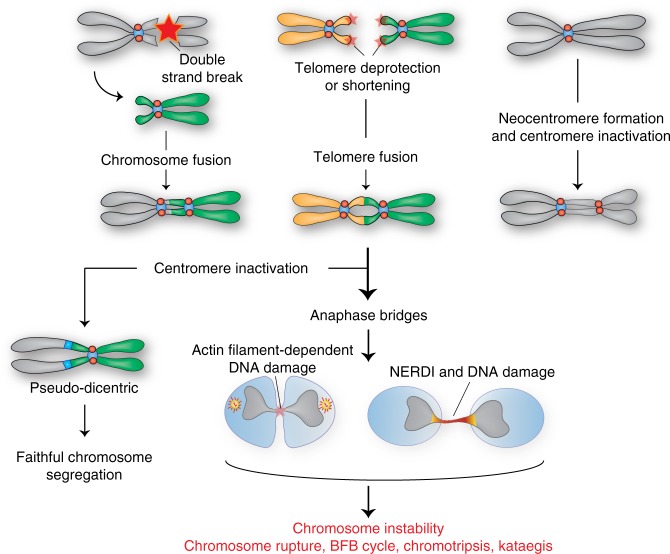
Dicentric chromosomes and chromosome instability. Representation of the possible origins of dicentric chromosomes that arise from chromosome/telomere fusions or neocentromere formation in the presence of a functional original centromere. During anaphase onset, the dicentric chromosome that is attached by opposing mitotic spindle fibers is trapped, leading to anaphase and chromosome bridges and, consequently, DNA damage and chromosome instability. Centromere inactivation could restore faithful segregation by preventing incorrect spindle attachment

The origin and fate of dicentric chromosomes have been acquiring remarkable importance in the past few years. Dicentric chromosomes were firstly described in maize by Barbara McClintock as the result of a type of crossing over between a broken chromosome and its normal homolog^136^. Indeed, it is believed that dicentric chromosomes arise after double-strand break formation leading to inversions and translocations or chromosome fusion via telomere deprotection (reviewed in ref. ^137^) (Fig. 4). In the case of chromosome rearrangements, a physical breakage of two chromosomes, homologs or not, can produce sticky ends that recombine end to end (inversion) generating one dicentric chromosome and two acentric fragments (one in the case of reciprocal translocation). Telomere shortening (e.g. during aging) or dysfunctional telomeres (e.g., loss of the protective Shelterin complex) can also generate telomeric fusions as observed during oncogenesis^138^ that, in turn, will generate dicentric chromosomes. Dicentric chromosomes can also originate in meiosis from U-Type exchange and NAHR (isodicentric chromosomes; as discussed in the section “Centromere breaks and disease”) and from Robertsonian translocations (see the section “Pseudodicentric chromosomes”)^41,139^.

### Consequences of dicentric chromosome formation

Dicentric chromosomes are genetically unstable during cell division because microtubules pull in opposite directions on the two centromeres of the same chromatid. This normally leads to the formation of chromosome bridges during anaphase, causing DNA breakage (see below). This phenomenon could potentially lead to cycles of BFB, as first documented by McClintock^140^.

In budding yeast the two centromeres of a single dicentric chromosome artificially induced by telomere HR and centromere silencing via active transcription^141^ were shown to attach to opposite mitotic spindle poles. Simultaneous pulling towards the opposite poles led to the formation of anaphase bridges^142^. Subsequently, dicentric chromosomes are broken at mitotic exit during cytokinesis in an actin filament-dependent mechanism (Fig. 4). The breakage site normally corresponds to the telomere fusion site in the case of dicentrics formed through telomere deprotection^143^ or to pericentromeric regions within a 25–30 kb range if the dicentric chromosome formation did not involve telomere fusion^142^. On the contrary, about 50% of dicentric chromosomes induced in human cells by manipulating a component of the shelterin complex appear to be (unexpectedly) stable after several cell cycles (up to 20 generations)^144^. Eventually, chromosome fragments containing mainly alpha-satellite DNA appear after several weeks. It is important to note that using this system, dicentric chromosomes were mainly generated between acrocentric chromosomes (up to 80% frequency), although stable dicentrics with an intergenic distance of more than 20 Mbs were observed^144^. Using a very similar approach in pRB/p21 negative human cells, dicentric chromosomes were shown to produce anaphase bridges that did not break during mitosis and persisted for 3–20 h after anaphase^145^. However, when extensively stretched, these long DNA bridges underwent transient nuclear envelope rupture during interphase (NERDI) giving access to cytoplasmic exonucleases such as TREX1. Nuclease activity led to ssDNA formation (indeed, 80% of these chromatin bridges accumulated RPA) favoring DNA repair and APOBEC-mediated editing of the fragmented chromatin bridge DNA. This chromatin fragmentation and repair was shown to generate chromothripsis and Kataegis (clusters of closely localized base substitutions)^145^ (Fig. 4).

Similarly, generation of a dicentric chromosome in humans by ectopic formation of a new centromere/kinetochore complex via CENP-T-LacI tethering to LacO gave rise to chromosome breakages. These breakages can be reduced by cytokinesis inhibition, suggesting that, at least in part, cytokinesis forces are involved in dicentric chromosome rupture^146^. The fragmented chromosome (of the dicentric chromosome) can in turn undergo chromosome fusions that result in new dicentric chromosomes recapitulating all the steps of the BFB cycle. Altogether, these processes/events will generate gene deletions, genetically unbalanced gametes and acquisition of features of transformed cells, such as invasiveness^146^.

Altogether, this suggests that the appearance of a dicentric chromosome could directly contribute and further exacerbate cellular transformation and cancer heterogeneity. Accordingly, stable dicentric chromosomes have been observed in some human tumors, such as in myeloid malignancies (myelodysplastic syndrome and acute myeloid leukemia), where they involve intercentromeric or centromere deletion, ring chromosomes and centromere inactivation (reviewed in ref. ^137^).

### Pseudodicentric chromosomes

Dicentric chromosomes are generally unstable (especially when generated experimentally), however there are cases of stable dicentric chromosomes, namely pseudodicentric chromosomes (Fig. 4). Their stability is conferred either by deletion of centromeric DNA sequence^144^ or (mostly) by epigenetic inactivation of one of the two centromeres, causing loss of centromeric components such as CENP-A and CENP-C^147,148^. An example of pseudodicentric chromosomes are those formed via Robertsonian translocations (ROBs) (Fig. 1). ROBs are a form of rearrangement involving vertebrates acrocentric chromosomes (13, 14, 15, 21, and 22), that mostly break at the pericentromeric region within satellite 3 DNA and that, through chromosomal fusion, generate dicentrics^149^. In the majority of cases, one of the two centromeres becomes inactive without deletion of centromeric DNA^150^. However, ROBs with functional dicentrics have been found^139^ most likely reflecting differences in the intercentromeric distances (reviewed in ref. ^151^). Two functional centromeres were also found in the dicentric chromosome X^152^ and in the isochromosome X in the case of selected somatic cell hybrids^153^. In some cases such as in ROBs in mice, the resulting fused chromosome originated from telocentric chromosomes gives rise to a metacentric chromosome following deletion of both telomeric and (part of) centromeric sequences (reviewed in ref. ^154^). Interestingly, the newly generated metacentric chromosome is stable and, in one documented case, it was even shown to be stably transmitted by increasing its centromere strength^155^. However, in humans carriers of ROBs have an increased risk of infertility since the resulting embryos can be un-viable or have elevated rates of Down Syndrome^156^. Even individuals that carry the most common ROB (13;14)—which constitutes 75% of all cases of this type of rearrangement and occurs in about 1/1000 new-borns^157^—have unfavorable pregnancy outcomes (miscarriages, chromosomal aberrations, stillbirth, and malformations)^158^, though whether they have an increased infertility rate is controversial^158,159^. Recently, this translocation has been correlated with developmental delay in a boy whose mother, also a ROB (13;14) carrier, was phenotypically healthy, suggesting a role of the (epi-)genomic environment in the outcome of the ROBs^160^. Carriers of the rare ROB (15; 21) show a 2700-fold increased risk of developing childhood acute lymphoblastic leukemia with intrachromosomal amplification of one copy of chromosome 21 (iAMP21 ALL). This high incidence correlated to the presence of rearrangements compatible with chromothripsis observed on the Roberstonian chromosome^161^. Other types of pseudodicentric chromosomes are Non-ROBs (in 85% of the cases involving one acrocentric chromosome) and are associated with several syndromes such as Kabuki syndrome, a genetic disorder with multiple congenital anomalies, intellectual disability, and growth deficiency^162^, Edward syndrome, a trisomy of chromosome 18^163^ and isodicentric X [i(X)] or Y [i(Y)] chromosomes such as Turner syndrome (as described in the paragraph “Centromere breaks and disease”).

## Misregulation of centromeric proteins

As observed for the dicentric chromosomes, the presence of centromeric components regulates centromere functionality. This is particularly true for CENP-A, the epigenetic marker that distinguishes the centromere from the rest of the chromatin. Although CENP-A is not exclusively found at centromeric regions, it is highly enriched at centromeres by ~40-fold compared to other regions in the chromosome and this accumulation is necessary to maintain centromeric identity^164^. For this reason both its expression and loading occur through a very tightly regulated process^165–168^. What are the consequences for the cells if CENP-A expression is deregulated?

On the molecular level, CENP-A overexpression (OE) leads to its massive delocalization outside the centromeres, mainly at regulatory elements^169,170^. Using chromatin immuno-precipitation, the Almouzni group demonstrated that promiscuous CENP-A incorporation is dependent on DAXX and occurs at CTCF (CCCTC-binding factor) locations and at sites with high histone turnover such as at H3.3 or H2AZ-rich regions or with H3K4me1 and H3K27ac post-translational modifications^169^.

Following precursor studies in yeast^170–172^ and flies^130^, Shrestha et al. showed that CENP-A OE and consequent mislocalization affected chromosome segregation in HeLa cells^173^. Interestingly, CENP-A OE altered CENP-C intensity at centromere and its localization, with CENP-C found at non-centromeric regions too. On the contrary, other centromere and kinetochore proteins, like CENP-T and Nuf2, were reduced following CENP-A OE but did not mis-localize. The authors suggested that this reduction contributes to a reduced inter-kinetochore distance that weakened the kinetochores and chromosome segregation fidelity, but without formation of ectopic kinetochores^173^. This finding is in agreement with what was previously observed by Van Hooser et al. where CENP-A OE was able to recruit a set of kinetochore proteins at ectopic sites that, however, were insufficient to form a fully functional kinetochore^174^, suggesting that centromere formation is regulated by multiple components. On the contrary, ectopic kinetochores following CENP-A OE can be observed in flies^130^. Interestingly, CENP-A overexpression in humans leads to increased tolerance of UV-induced DNA damage^169^. This suggests that acentric chromosome fragments that arise following DNA damage can be preserved in the cells by forming functional centromeres due to CENP-A enrichment.

CENP-A expression might be regulated by tumor suppressor genes such as pRB (Retinoblastoma protein)^175^. Interestingly, pRB depletion, frequently found mutated in human cancers, correlates with compromised chromatids cohesion and centromere distortion that negatively compromise mitosis^176^. It is likely that a fraction of these mitotic defects can be directly attributed to CENP-A overexpression, since restoration of CENP-A levels by siRNA prevents micronuclei formation and aneuploidy^175^ and prevents hepatocellular carcinoma growth^177^. Indeed, increased levels of CENP-A and its chaperone HJURP^178,179^ are found in several tumors like hepatocellular carcinoma, breast cancer and colorectal cancer as described above^129,177,180–187^. In fact, a comprehensive study on public gene expression data sets of normal and cancer tissues (HBI, expO, and CCLE) revealed that core kinetochore and cell cycle genes were significantly up-regulated in tumors. Interestingly, the authors show that upregulation of these genes correlates with high levels of Forkhead Box M1 (FoxM1), a transcription factor known to bind the promoter of the majority of kinetochore genes and cell cycle players^188^. Accordingly, a more recent bionformatics analysis on 13 data sets from 12 different types of human cancer in the Gene Expression Omnibus (GEO) database, revealed that there is a progressive increase in centromere and kinetochore gene expression (Centromere and kinetochore gene Expression Score, CES) during disease progression of breast, prostate and liver cancers^189^. These findings suggest that it is possible to draw a gene expression signature predicting cancer patient outcome based on CES. Interestingly, CENP-A is overexpressed in almost all the analyzed cancers, independently of the progression stage, suggesting that this alteration could contribute to cell transformation. More precisely, Filipescu et al. found that CENP-A is overexpressed in p53-null tumors and that CENP-A (and HJURP) OE is a consequence of p53 inactivation since it occurred in MEFs where p53 function was suppressed^190^. They also showed that HJURP OE is necessary to sustain growth of p53-null transformed cells due to an increase in proliferation versus normal cells.

On the contrary, CENP-A reduction goes in parallel with cell senescence and aging: CENP-A abundance decreases in cells during aging and it becomes almost undetectable in human islets after 29 years of age^191^. CENP-A reduction by shRNA in an in vitro tissue culture system blocks cell cycle progression in G1 in a p53-dependent manner (likely in response to aneuploidy) leading to either premature senescence^192^ or activation of the apoptotic pathway^177^.

Similarly, reduction of HJURP causes a decrease in cell proliferation that correlates with p53 and p21 stabilization^193^. This cell cycle block is likely to occur in response to aneuploidy, although if this is the only consequence of p53 activation was never directly demonstrated. For example, Giunta and Funabiki described that CENP-A-depleted cells and senescent cells undergo centromeric aberrations likely due to an imbalance in DNA recombination at centromeric regions^92^. This increase in recombination could generate centromere erosion due to shortening of repetitive DNA. Intriguingly, centromere deterioration was observed during aging^194^. Senescent cells are also subject to several chromatin changes such as variations in the degree of compaction of pericentromeric/centromeric regions named senescence-associated distension of satellites (SADS). This change was proposed to be an early event that would mark senescent cells^195^.

The amount of centromeric components at centromeric regions might also have an impact on the frequency of chromosome mis-segregation, a concept defined as “centromere strength”. A good example of this concept is the Y chromosome, that not only is completely devoid of CENP-B boxes and CENP-B, known to support centromere function, but also contains the shortest alpha-satellite array of all chromosomes^196^. This CENP-B-free human chromosome has reduced levels of some centromeric components such as CENP-A^197^ and CENP-C^75^ and mis-segregates at elevated frequencies^12^. Intriguingly, the Y chromosome was found to mis-segregate at higher rates in an age- and smoke-dependent manner (2.4 to 4.3 fold increase) and its loss correlates with high risk of blood cancer and shortened survival (50% probability of survival)^198–200^.

## Concluding remarks

Centromeres are the fundamental units of chromosome inheritance. Nevertheless, DNA breaks, rearrangements and structural aberrations at centromeric regions are commonly observed in cancer cells and some genetic syndromes. The causes underlying this intrinsic fragility are still untested.

The repetitive nature of centromeric DNA sequences might provide a favorable environment that helps maintain centromere position and assembly. This in turn sustains faithful chromosome segregation, ultimately contributing to preserving genome integrity. Given the recurrence of repetitive DNA in centromeres throughout evolution it has been suggested that the centromeric sequences were naturally selected more for the ability to form a particular structure than for the DNA sequence itself. The resulting highly organized region could play a role in supporting the mechanical stress generated by the spindle microtubules pulling on the centromere/kinetochore platform during chromosome segregation. However, maintaining repetitive DNA also has major drawbacks. Repetitive sequences are generally unstable and might form secondary structures that could potentially induce replication fork stalling, topological problems, and high levels of recombination. Altogether, these anomalies could lead to DNA breakage in certain genetic backgrounds. Indeed, due to the repetitive nature of their DNA sequences, human centromeres are proposed to be enriched with DNA catenates and loops between their DNA repetitive arrays. A well-defined and organized machinery would then be required to solve those structures prior to segregation in order to avoid centromere breaks. In addition, centromeres might also be difficult to replicate leading to an accumulation of replication fork stalling and collapse (especially since centromeres are commonly late replicating regions), which could result in double-strand breaks. It seems that the centromeric structures themselves are necessary to activate unique pathways essential to complete DNA replication prior to chromosome segregation. Curiously, it was proposed that some components normally present during DNA replication such as RPA-1 and ATR are excluded from centromeric regions but become essential during mitosis for correct chromosome segregation. Altogether, this highlights the uniqueness of centromeric regions and how changes in these intricate mechanisms can lead to errors and breakage.

Centromere alterations such as the appearance of neocentromeres and dicentric chromosomes generated by chromosome rearrangements are also found in disease and most of the time, once formed, are remarkably stable during both mitosis and meiosis. While more is known about how dicentric chromosomes form and contribute to genome instability, the generation and the role of neocentromere as a source of instability is still not fully explored. This is why models of de novo centromere formation coupled with centromere inactivation are extremely valuable to understand neocentromeres genesis and its pathological outcome. In addition, varying the expression levels of centromeric components, first and foremost CENP-A and its chaperone HJURP, has been shown to have opposite effects on cells: while downregulation promotes both cell proliferation arrest and senescence, upregulation leads to chromosome instability and cancer transformation, although via molecular mechanisms that need to be clarified; indeed, CENP expression levels could now be used as biomarkers for some types of cancers.

In conclusion, centromeres can be the subject of alterations that contribute to chromosome instability and that are found in human diseases. To what extent and at which stage centromere dysfunctions directly participate to these pathological events is still unclear. For this reason, models and technologies to induce and assess centromere alterations are needed. Until now, centromere analysis was largely limited by the lack of appropriate DNA sequencing technologies for such large repetitive sequences, making centromere sequences the black hole of the human genome. Because of these technical limitations, it is still unclear if the chromosomal breakpoints observed at centromeric regions are actually at the centromeres per se or at the surrounding pericentromeric regions rich in heterochromatin. The advent of more sophisticated technologies will be essential to reveal instability within these regions in human disease to finally shed light on the dark side of the centromeres.
